# Tophaceous Gout – Even Today

**DOI:** 10.3389/fimmu.2013.00284

**Published:** 2013-09-23

**Authors:** Elena Polukhin, Jaya Durvasula

**Affiliations:** ^1^Rehabilitation Consultants PA, Eagan, MN, USA

**Keywords:** gout, gout suppressants, chronic disease, chronic pain, tophaceous gout

Gout is a relatively rare disease that generally occurs in the middle-aged population. It is usually mild, well controlled using modern medication, and rarely causes significant joint deformities. Occasionally, the clinical course of gout might be severe, leading to severe joint deformity, chronic pain, and prominent functional decline. However, the disease is more pronounced in Asian patients; so-called “slow metabolizers,” and in young men with a strong genetic predisposition.

Here is the story of a 30-year-old Asian patient with a family history of Gout. He has suffered from severe gout beginning as a teenager, and continuing for nearly two decades. He is a 30-year-old Hmong male who arrived at our clinic with complaints of chronic pain, severe feet and hands deformity, and history of multiple gout flares. Other than that, the patient was healthy.

The patient’s father’s brother suffered from gout as well, but had less prominent joints deformities. The patient’s mom has a long history of nephrolithiasis. She had undergone surgical removal of about a 2′ long renal calculus once.

**Figure 1:**
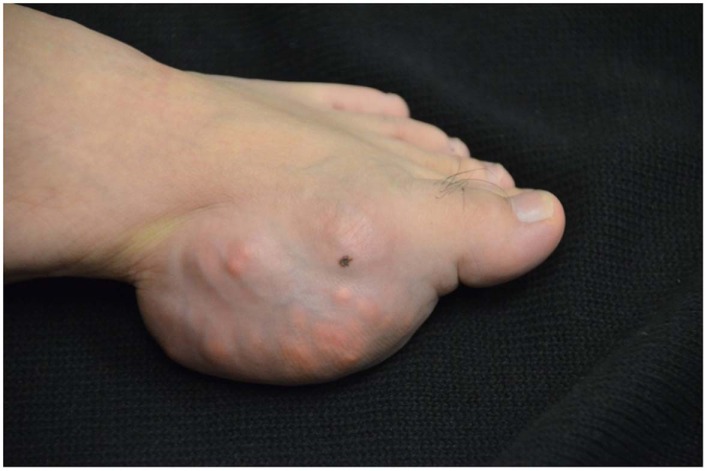
**Severe feet deformities and tophi accumulation**.

The patient first had pain in his ankles at age 16. At the time he was an avid athlete and attributed joint pain to the sports injury. The pain subsided after Tylenol and Ibuprofen, but he continued having monthly intermittent attacks of swelling, redness, and severe pain in his feet, then his ankles, and then his hands. Gradually, he started having swelling and tophi in the first metatarsophalangeal joints of both feet. His left foot is shown in Figure 1. Tophi then started in the second and fourth metacarpophalangeal joints, appearing later on in his elbows, and in his knees. He presented with excruciating joint pain to the E.R repeatedly, and finally he was diagnosed with gout.

He was advised to have a preventive gout treatment, but because of the lack of medical insurance he had neither treatment, nor prophylaxis. The patient attempted to get medical insurance, but because of the pre-existing condition, his application was denied by many insurance companies and he was unable to afford a high deductible of an alternative insurance. Finally, he applied to our clinic for *pro bono* care, was treated with modern medications (Uloric and Colcrys). His condition significantly improved, pain subsided, and he was able to continue his personal life and professional career.

Since this patient lacked adequate health-care coverage, we decided to treat him on a *pro bono* basis at our clinic in Minnesota. At present, Minnesota has embraced the Affordable Health Care Act more ambitiously than any other state. We expect that 160,000 more Minnesotans will receive health insurance by 2014.

We also see universal health coverage being evaluated at the international level with the World Health Organization’s stated goal “to ensure that all people obtain the health services they need – prevention, promotion, treatment, rehabilitation, and palliation – without risk of financial ruin or impoverishment, now and in the future.” Among the findings by WHO in its World Health Report 2013 “Research for Universal Health Coverage” was that conditions causing ill-health, and the financial capacity to protect people from ill-health, vary among countries, and that each nation must determine its own priorities: World report 2013, Research for universal health coverage, World Health Organization, 2013.

While we applaud our efforts in our own backyard, and look forward to the much anticipated results of hard fought political battles in health-care reform, we as physicians will need to continue to wear many hats including that of the provider of life-saving services to those in need, the voice of reform and at times the hat of the politician.

We hope that Affordable Care Act will bring an end to health-care disparities in our country and allow sick people equal access to affordable medical care. It will prevent severe debilitating cases like this one and development of chronic disease complications.

